# Predicting Bullying through Motivation and Teaching Styles in Physical Education

**DOI:** 10.3390/ijerph17010087

**Published:** 2019-12-21

**Authors:** Carlos Montero-Carretero, David Barbado, Eduardo Cervelló

**Affiliations:** Department of Sport Sciences. Sport Research Center, Miguel Hernández University, 03202 Elche, Spain; dbarbado@goumh.es (D.B.); ecervello@goumh.es (E.C.)

**Keywords:** teaching support, basic psychological needs, motivation, physical education, bullying

## Abstract

From the theoretical framework offered by the self-determination theory, the objective of the study was to test a predictor model of bullying behaviors based on the physical education teacher’s supportive style, the students’ satisfaction of basic psychological needs, and self-determined motivation. A total of 608 students of both sexes, between 11 and 15 years of age, from primary and secondary schools in the province of Alicante (Spain) voluntarily completed questionnaires to measure each of the variables under study. The design of the study was cross-sectional. The results showed that the autonomy supportive style positively predicted the satisfaction of basic psychological needs, which, in turn, positively predicted self-determined motivation towards physical education. The latter negatively predicted bullying perpetration and bullying victimization. The controlling style presented inverse relationships to those of the autonomy supportive style. These results are in line with the positions of the self-determination theory and underline the potential responsibility of physical education teachers in the struggle against bullying, and how, by supporting autonomy and avoiding a controlling style, they can help reduce bullying perpetration and victimization.

## 1. Introduction

Bullying is one of the main problems faced by the educational community. The school is a unique microsystem [1] in which teachers have special influence over the students’ relationships and behaviors, and wherein the subject of physical education (PE) could have a specific weight in the development of bullying behaviors [2]. Although the self-determination theory (SDT) [3,4] has proved to be very useful in explaining the behavior of human beings based on their motivations, little research has explained bullying from this theoretical framework, let alone from the context of PE.

Understanding the relationships between the teacher’s supportive style, students’ motivations in PE, and bullying, would be useful to develop effective prevention strategies for such behaviors.

### 1.1. Bullying

Bullying has been defined as an aggressive behavior that is repeated over time, with the intention of causing physical, psychological, social, or educational harm, where there is an imbalance of power between aggressors and victims, who are not siblings or current dating partners [5]. Victims can be beaten, insulted, threatened, socially excluded, or suffer damage to their property, often in the presence of spectators, who take on multiple roles depending on their attitude toward the bullying event [6].

Bullying is alarmingly present in schools in Spain and the rest of the world [7,8], both because of the number of students involved, and because of the devastating consequences that it entails. Victims of bullying present mental health problems, including eating disorders, low self-esteem, loneliness, poor relationship quality, self-harm, anxiety, symptoms of depression, and suicidal thoughts that are sometimes enacted upon [9,10]. Bullies also suffer from mental health problems such as anxiety, depression, psychosomatic problems, an increased risk of suicide, future relationships with other forms of violence, and risk of excessive alcohol and marijuana use [11,12]. Finally, spectators are not exempt from the consequences, sometimes suffering from distress, anxiety, and depression [13]. 

### 1.2. Physical Education and Bullying

Students who engage in intimidating behavior do so in an attempt to acquire greater status and a position of power within the group [14]. This end is often achieved at the expense of students with physical weaknesses, a lack of skill in the development of motor tasks [15], and/or differences in their physical constitution, such as obesity or being overweight [16]. In this sense, the subject of PE can particularly expose the most vulnerable students in the eyes of their potential aggressors, both because of the characteristics of the spaces where PE is practiced [2] and because of the subject’s demands of public demonstrations. In PE, young people relate to their peers in a very different way from how they relate in other subjects, and there is usually physical contact in PE. Thus, instead of being able to move away from their aggressors, victimized students are forced to interact with them [2]. This reasoning is supported by a study of American students [17], in which it was observed that bullying victims had more hours of PE than non-victimized students, suggesting that PE classes could be a critical space for the development of bullying behaviors. However, these classes could also be an ideal means of detecting and addressing bullying situations [15].

Despite these results, several authors do not support this theory. Roman and Taylor [18] argue that school environments that do not induce physical activity favor bullying behaviors. This seems to be supported by the fact that low levels of physical activity and sedentary behavior are linked to victimization [19]. In addition, PE is important in the acquisition of healthy behaviors related to the practice of physical activity in leisure time, which has been linked to students’ better academic performance and health [20,21].

### 1.3. Self-Determination Theory (SDT), Physical Education, and Bullying

To explain students’ motivations towards PE from SDT [3,4], it is postulated that, influenced by close social actors, people strive to meet three basic psychological needs (BPN; autonomy, competence, and relatedness) in order to experience healthy growth and high levels of well-being. Students’ autonomy is defined as their perception of their ability to make decisions and choose freely. Competence refers to how skillful or good students feel about the tasks that are demanded of them. Finally, relatedness represents the importance of feeling connected to the people in their class, integrated within the group, well-regarded and well-treated by peers and teachers [22]. Students’ motivations towards PE will depend on the degree of BPN satisfaction in PE classes.

Vallerand et al. [23] propose a seven-dimensional continuum which, from more to less self-determined, consists of three types of intrinsic motivation (IM: to learn, to improve, and towards stimulation), four types of extrinsic motivation (EM: integrated, identified, introjected, and external regulation), and amotivation. The level of self-determination refers to the degree to which the reasons for manifesting a behavior, for example, participating in PE classes, are more internal or external, as well as their voluntariness. Thus, students sometimes participate in PE classes for intrinsic reasons, to learn things related to the subject, to improve their motor skills, or because of the stimulating feeling that it provokes. However, sometimes, motivations for participating in PE are not inherent to PE. This is the case with extrinsic motivations such as integrated regulation, which occurs when students participate in PE because their own values are in harmony with those of that subject. Identified regulation occurs when students participate in PE because they positively identify what this entails. Introjected regulation occurs when the main reason for participating is to avoid guilt. Finally, external regulation is typical of those who participate in PE to avoid punishments or to obtain rewards. When people do not find a satisfactory relationship between the effort demanded and the reward of participating, they are considered unmotivated (amotivation). Unmotivated students tend to have difficulty adjusting to school, are less persistent in completing their studies, and are less satisfied with their academic experiences [24]. These results support the tenets of the SDT. According to these tenets, less self-determined motivations lead to unadaptive consequences, while more self-determined motivations lead to adaptive consequences. These consequences can be cognitive, affective, or behavioral. Thus, in the context of SDT, bullying could be considered as an unadaptive behavioral consequence.

Regarding bullying analyzed from the SDT, Roth and Bibi [25] found that the internalization of prosocial values due to identified regulation negatively predicted bullying, whereas the external regulations of those values predicted it positively. Subsequently, Goodboy et al. [26] showed that those who suffered bullying in high school presented lower levels of self-determined motivations (introjected and external regulation), high levels of amotivation, as well as academic, social, emotional, and institutional problems in their first semester of college. On the contrary, works such as that of Jungert et al. [27] showed that students who presented higher levels of self-determined motivations towards the behavior of victim defense helped more in the face of bullying events than students who presented lower levels of self-determined motivations.

Regarding the teacher’s supportive style, SDT indicates the importance of social agents who are close to the students as determinant in the satisfaction of their BPNs. Reeve [28] states that, in PE classes, the most prominent source of support for students’ needs is the teacher’s motivating style.

Among the different supportive styles presented by teachers in their classes, the most frequently studied have been autonomy support (AS) and the controlling style (CS). Referring to AS, “contexts of support for autonomy involve the recognition of the child’s feelings, the adoption of the child’s perspective, justification of decisions made by teachers, the possibility of choice and the minimization of pressure” [29] (p. 656). For their part, Guay and Vallerand [30] (p. 215) define the teacher’s AS as “the degree to which people use techniques that encourage choice and participation in school activities”. AS has been linked to higher levels of self-determined motivation in PE students [31], positively influencing affective relationships, student behavior in class [32], and prosocial behavior [33] and a stronger intention to be physically active [34]. Tilga et al. [32] propose that autonomy-supportive behavior could be characterized by three dimensions, namely organizational, procedural, and cognitive. Thus, organizational autonomy support encourages students to take possession of the environment and could include teaching behaviors that offer students opportunities to choose the teaching methods and where to perform an exercise. Procedural autonomy support encourages students to become the owners of the way activities are performed and could include teaching behaviors such as offering students the choice of how to present homework. Cognitive autonomy support encourages student learning and could include teaching behaviors such as asking the students to justify and defend their point of view, or to seek solutions to a problem.

Regarding bullying from the viewpoint of SDT, [29] observed an inverse relationship between the AS perceived by students and bullying behavior, mediated by the internalization of consideration towards their peers. Lam et al. [1] recommend AS styles for the promotion of BPN satisfaction in PE, noting in their study that AS style negatively predicted bullying behaviors, based on the satisfaction of the need for relatedness.

CS is characterized by teachers’ authoritarian attitudes, ignoring the students’ perceptions, and pressing to impose a specific and preconceived way of thinking, feeling, and behaving [28,35]. There are two distinct ways through which the teacher tries to exercise control: external and internal. As external sources of pressure, teachers use aggressive behaviors based on screams, threats, or attacks on some students to disparage them. In PE, physical punishments, such as doing push-ups or running in the playground, are imposed [35]. Internal control is shown when teachers try to provoke feelings of guilt or shame in the student, withdrawing their attention or interest and expressing disappointment when their expectations are not met [36], which generates anxiety and harms the student’s self-esteem.

CS has been linked to low levels of perceived satisfaction and student engagement [37], increase in levels of physiological stress markers such as cortisol [38], and increases in anger and anxiety [39]. A recent study in PE [40] showed that CS predicted BPN thwarting, controlled forms of motivation, and amotivation. These, in turn, predicted fear of failure, low self-esteem, and avoidance of challenges. CS in PE was also significantly associated with BPN thwarting, bullying, and anger, primarily through intimidation [41].

### 1.4. The Present Study

Several authors recommend considering the context of PE in the development of bullying behaviors [2,15], as it can affect bullying perpetration and victimization predictors, such as the level of physical activity or more or less healthy habits [42].

However, although the importance of teaching styles in the satisfaction/thwarting of students’ BPN [1,40] and their motivations [31,43] has been highlighted, very few studies have explained bullying from the SDT viewpoint, and only a few have linked types of motivation to bullying perpetration and victimization [25,29]. To our knowledge, no studies have analyzed the effect of teachers’ supportive style on students’ BPN satisfaction and motivation in PE to explain the emergence of bullying behaviors. Therefore, the main objective of this study was to test a predictive model of bullying, from the SDT perspective in the PE context, which included these variables.

Based on the results of previous work, it was hypothesized that:

1. Teachers’ AS styles would positively predict BPN satisfaction in PE [1], whereas CS would predict it negatively [40,41].
2. BPN satisfaction in PE would positively predict self-determined motivation towards PE, in line with the SDT postulates [3,4] and many prior studies [42,44]. Self-determined motivation is expected to be directly predicted by teacher supportive styles, as was the case in previous studies [27].
3. Self-determined motivation in PE would negatively predict bullying perpetration and victimization, considering the above-mentioned relationships between self-determination and bullying [25,29].
4. Victimization would positively predict bullying perpetration, considering that many victimized students subsequently become bullies [1,12,45,46].
5. AS in PE would have indirect negative effects on bullying perpetration and victimization [29], whereas CS would have indirect positive effects on such bullying behaviors [41].

## 2. Materials and Methods

### 2.1. Particpants

A cross sectional design was employed to complete the study. Participants in the study were 608 students (Mean ± SD: 12.49 ± 0.98 years old; 308 girls and 300 boys) from 8 schools (5 public and 3 subsidized) in the province of Alicante (Spain). Regarding the grade, 163 were in 6th grade of Primary School, 247 were 1st-grade students of Compulsory Secondary Education (CSE), and 198 were 2nd-graders of CSE. The sample was a convenience sample. The participating schools were chosen by the competent educational authority responsible for specific schools. In fact, the authorization file indicated this. We measured in the age groups of our interest in all those schools from which we received authorization.

### 2.2. Measuring Instruments

The following measuring tools used to analyze the study variables:

Measure of Teachers’ Supportive Style: “Escala de Percepción del Estilo de Soporte en las Clases de Educación Física” (EPES-PE (Scale of Perception of Supportive Style in Physical Education Classes)) was used. This scale [47] is an adaptation of the Multidimensional Perceived Autonomy Support Scale for Physical Education (MD-PASS-PE) of Tilga et al.’s [32] scales to measure AS, and part of the Empowering and Disempowering Motivational Climate Questionnaire (EDMCQ-C) of Appleton et al. [48] to measure CS. It consists of 19 items grouped into 4 correlated latent factors that have shown adequate fit indices [47]: (a) Organizational support autonomy (5 items), (b) Procedural support autonomy (5 items), (c) Cognitive support autonomy (5 items) and Teacher’s CS (4 items). Responses are rated on a seven-point Likert scale ranging from 1 (totally disagree) to 7 (totally agree).

Items related to Organizational autonomy support describe situations such as: “My PE teacher allows me to exercise in different ways”. Those related to procedural autonomy support describe situations such as: “My PE teacher helps the students to find solutions”. Cognitive autonomy support items describe situations such as: “My PE teacher takes into account what the students want to do”. Finally, the CS items describe situations such as: “My teacher is less friendly to students who don’t see things his/her way”.

A general measure of AS can also be calculated, which is recommended when performing mathematical prediction processes [32] to avoid problems of collinearity and variance inflation. In our study, the alpha coefficients were 0.79 for the CS factor, and 0.80 and 0.92 for all three AS factors, with the global AS factor obtaining a value of 0.94.

Measure of BPN Satisfaction: The Spanish version of the Basic Psychological Needs in Exercise Scale [49], adapted to the context of PE by Moreno et al. [50], was used. The scale was preceded by the statement “In the subject of physical education...” and was composed of 12 items, 4 for each of the factors: autonomy (e.g., “The exercises I carried out matched my interests”), competence (e.g., “I performed the exercises effectively”) and relatedness (e.g., “I felt very comfortable with my classmates”). Responses were rated on a five-point Likert scale ranging from 1 (totally disagree) to 5 (totally agree). The alpha coefficients in our study were 0.78 for Autonomy Satisfaction, 0.83 for Competence Satisfaction, and 0.78 for relatedness satisfaction. The global factor obtained an alpha value of 0.90.

Measure of Motivation in Physical Education clases: To measure students’ self-determined motivation, the physical education motivation questionnaire (CMPE) validated by Sánchez-Oliva et al. [51] was used. This scale is composed of the stem phrase “I participate in physical education classes…”, followed by 20 items that analyze the five factors: intrinsic motivation (4 items, e.g., “Because physical education is fun”), identified regulation (4 items; e.g., “Because this subject provides knowledge and skills that I consider important”), introjected regulation (4 items; e.g., “Because I think it is necessary to feel good about myself”), external regulation (4 items: e.g., “To show my interest in the subject to the teacher and peers”), and amotivation (4 items; e.g., “But I really feel like I’m wasting my time with this subject”). Participants had to express their degree of agreement on a five-point Likert scale ranging from 1 (totally disagree) to 5 (totally agree). In our work, the alpha values were 0.85 for intrinsic regulation, 0.86 for identified regulation, 0.79 for introjected regulation, 0.79 for external regulation, and 0.85 for amotivation.

The self-determination index (SDI) was calculated based on the dimensions of the questionnaire [43]. SDI = (2 × intrinsic motivation + identified regulation) − [(introjected regulation + external regulation)/2 + 2 × amotivation]. The higher the score in this index, the more self-determined is the individual’s motivation. In our study, reliability ranged from −11.38 to 11.50.

Measurement of Bullying: The Spanish version of the European Bullying Intervention Project Questionnaire (EBIP-Q) of Ortega-Ruiz et al. [52] was used to measure this variable. This scale includes two factors, which reflect bullying victimization and bullying perpetration, with 7 items each. The first 7 items are related to victimization, describing situations such as: “Someone has stolen or broken my things; Someone has threatened me; Someone has insulted me”. The last 7 items are related to Bullying Perpetration, describing situations such as: “I’ve stolen or ruined someone’s things; I’ve threatened someone; I’ve spread rumors about someone”. Students are asked to indicate how often they have performed or suffered these behaviors in the past two months. Each item is presented as a direct sentence in the first person. The student must answer them on a five-point Likert scale, as follows: 1 (No), 2 (yes, once or twice), 3 (yes, once or twice a month), 4 (yes, about once a week) to 5 (yes, more than once a week). The alpha values in this work were 0.83 for the Bullying Perpetration factor and also 0.83 for the Victimization factor.

### 2.3. Procedure

After obtaining the relevant authorizations (centers, parents, and Autonomous Secretariat of Education—file 05ED01Z/2017.56), we scheduled the day to conduct the surveys with the teachers in charge. The school directors were then contacted to encourage them to participate and inform them about the objectives of the study, as well as its exclusively scientific and academic purposes. In addition, they were informed of the voluntary nature of the test, and the strict confidentiality of the data obtained therein. Once the school directors had agreed, a written statement was sent to request the informed consent of the parents.

Data collection was carried out in a classroom of each school in one of the classes scheduled for PE during the first trimester. Prior to the test, students were instructed about the importance of responding sincerely. During the completion of the questionnaires, any doubts that arose were clarified by the teacher of the subject. The questionnaires were completed in approximately 20 min.

## 3. Results

### 3.1. Statistical Analysis

Descriptive statistics (means and SD), Pearson correlations, and path analysis were employed to develop the results. The differences by gender and type of School (public versus subsidized) was analyzed using a Manova (multiple analysis of variance). The maximum likelihood estimation method was used for path analysis and, following the recommendations of Hu and Bentler [53], a combination of several fit indices was used to contrast the adequacy of the proposed models. Specifically, the ratio between chi squared and degrees of freedom (χ^2^/df), the comparative fit index (CFI), incremental fit index (IFI), root mean square error of approximation (RMSEA) plus its 90% confidence interval (CI), and the standardized root mean square residual (SRMR) were used. For the χ^2^/df coefficient, values below 3 are generally considered acceptable, although some more conservative authors accept values below 5 [53]. RMSEA values equal to or less than 0.08 and SRMR values equal to or less than 0.06 are considered acceptable. However, there is a widespread consensus to consider that these values are only indicative [54], and that for indices such as the CFI and IFI, more conservative criteria (values equal to or greater than 0.90) are considered an acceptable lower limit. In short, rather than a single value, a global exploration of all values must be performed to accept the proposed model.

### 3.2. Descriptive Statistics and Correlation Analysis and Differences by Gender and Type of Center

Table 1 shows the values of the descriptive statistics and the correlations of the study variables.

Regarding correlations, we observed a positive correlation between the teacher’s AS and students’ satisfaction of BPNs and self-determination index. AS also correlated negatively with bullying perpetration and victimization. On the contrary, the perception of CS correlated negatively with the satisfaction of BPNs and the self-determination index, and positively with bullying perpetration. The self-determination index correlated negatively with bullying perpetration and victimization. Lastly, we note the positive correlation between bullying perpetration and victimization (Table 1).

No differences were obtained on the analyzed variables for gender (Wilk’s Lambda = 0.982; *p* < 0.89) type of center (Wilk’s lambda = 0.973; *p* < 0.310) and interaction between gender and type of center (Wilk’s Lambda = 0.998; *p* < 0.935).

### 3.3. Prediction of Bullying Perpetration and Bullying Victimization Based on the Perception of the Physical Education Teacher’s Supportive Style, the Satisfaction of BPNs in Physical Education Class, and Students’ Self-Determination in the PE Class.

**Figure 1:**
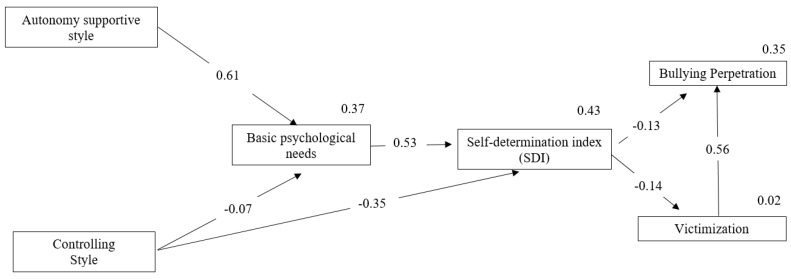
The final standardized solution of the teacher’s supportive style prediction model, BPN satisfaction in physical education, self-determination index, and bullying perpetration and victimization. (Only statistically significant paths are presented, with regression weights appearing over the lines, and the explained variance over the variables).

To study how the physical education teacher’s supportive style, BPN satisfaction, and the self-determination index all predict bullying, a path analysis with the Amos 19 de IBM SPSS software was performed following the guidelines of Hu and Bentler [53], and including only those paths that showed significant predictions (Figure 1). The analysis showed good fit indices both for incremental fits and error indices (χ^2^ = 34.23; χ^2^/df = 4.27; CFI = 0.97; IFI = 0.97; SRMR = 0.05; RMSEA = 0.07). The direct effects showed that AS positively predicted satisfaction of BPNs, whereas CS negatively predicted BPN satisfaction. The satisfaction of BPNs positively predicted the self-determination index and this, in turn, negatively predicted both bullying perpetration and victimization. Finally, CS directly and negatively predicted the self-determination index, whereas victimization positively predicted bullying perpetration.

Regarding indirect effects, we found indirect negative effects of AS on bullying perpetration (−0.044) and victimization (−0.066), whereas CS positively predicted bullying perpetration (0.078) and victimization (0.053).

## 4. Discussion

Considering that little research has explained bullying from the perspective of SDT [3,4], the main objective of the work was to evaluate, from this theoretical framework, a predictive model of bullying perpetration and victimization based on PE teachers’ supportive style, BPN satisfaction, and student motivation for this subject.

The results show that student-perceived AS positively predicted their BPN satisfaction, whereas CS did so negatively, confirming Hypothesis 1. In addition, in favor of Hypothesis 2, BPN satisfaction positively predicted students’ self-determined motivation in PE, whereas CS predicted it negatively, as was the case in previous studies [34]. These data reinforce the importance of teachers’ training, not only to increase their students’ satisfaction of BPNs, but also to promote self-determined motivations through non-authoritarian styles, where teachers justify their decisions, facilitate students’ choices, and minimize pressure on the students [29,30]. Although satisfaction of the need for relatedness in PE negatively predicted bullying perpetration [1], whereas BPN thwarting positively predicted it [41]. To our knowledge, there is no work to date showing the predictive nature of motivation in PE and the emergence of bullying behaviors. Based on this and confirming Hypothesis 3, the fact that self-determined motivation towards PE negatively predicts bullying perpetration and victimization is a relevant contribution to the treatment of bullying. These results are in line with previous works [25,29], which indicated the importance of acquiring prosocial values for self-determined reasons to prevent bullying behaviors in the academic context.

In this investigation, victimization predicted bullying perpetration, confirming Hypothesis 4, which shows that victims are in serious danger of becoming bullies [1,12,45,46]. For this reason, schools should establish protocols of attention to victim that, in addition to repairing the harm suffered from bullying, will prevent the victims from intimidating others in the future.

Finally, the indirect negative effects of AS on bullying and the positive effects of CS on bullying behaviors confirmed Hypothesis 5. These results support the importance of the teacher’s style for students to acquire prosocial values for self-determined reasons, internalizing and integrating them into their own code of ethics [29]. In this vein, a study conducted in PE [44] showed that a teaching style based on promoting BPN satisfaction predicted students’ positive behavior regarding peer respect, self-control, and cooperation through BPN satisfaction and self-determined motivations.

Our findings provide some support to both studies [29,44], showing the effect that PE teacher supportive style could have on preventing bullying behaviors through BPN satisfaction and self-determined motivations towards this subject. Roth et al. [29] argue that styles based on controlling student behaviors could be inefficient in preventing the occurrence of bullying perpetration and victimization, as they would help students to avoid performing such behaviors publicly in environments where they feel controlled, but not when they feel unprotected by the faculty’s surveillance. Therefore, PE teachers are encouraged to avoid controlling their students’ behaviors through punishments, public reprimands, or withdrawal of attention when the students do not follow their instructions exactly, or by expressing discontent due to frustrated expectations. Such strategies seem inadequate for the promotion of BPN satisfaction [40,41], and could contribute to generating patterns and mechanisms to avoid performing undesirable behaviors only when under supervision. On the other hand, considering the findings about AS, it seems appropriate for teachers to promote students’ BPN satisfaction through various strategies: expressing closeness, concern about their problems, providing them with the possibility to choose some tasks to perform, task order, the classmates with whom to work, the places where they perform the tasks, the time spent to do each one, or how to do them to achieve the proposed goal. However, it is important that the rules about bullying are clear, and that the teacher is assertive in certain situations, providing spaces where potential victims feel safe [55] to reduce victimization.

Finally, considering the particularities of PE [2], teachers could empower students who exhibit particular characteristics because of their physique or apparent lack of motor ability, trying to help them advance gradually and developing achievable goals for them, placing their strengths at the service of the group and recognizing their progress. For example, students who are somewhat overweight—often victimized for this reason—might take on force-building tasks such as carrying their classmates when performing sports exercises such as acrosport, or adding points for the group in judo games where the goal is to hold the opponent face-up for a set time under safety rules, etc. In contrast, those who show apparent weakness could stand out and contribute in other roles within these or other games, for example, showing the ability to develop strategies and responses to problem solving. It is the teacher’s job to detect students’ skills in order to help them stand out through using them, and to avoid constantly exposing them for their weaknesses. Collaborative and teamwork games, together with teacher-managed reflections on diversity, tolerance, and respect, could bring potential victims closer to the peer group, strengthening ties with each other, making it difficult for potential bullies to obtain reinforcement for aggression, and thwarting the goal of gaining social recognition through intimidation.

## 5. Limitations and Future Prospects

Obviously, this study is not exempt from limitations. We note among the main ones the exclusive use of self-reports for data collection, the cross-sectional nature of the work, and the fact of testing a single model and in just one sample. In this study, the variables were measured at a single moment, which is a methodological limitation. Accordingly, we propose that future studies replicate with other samples the analysis of the model presented using longitudinal designs, measuring at two or three different moments. We consider as a limitation the exclusive use of correlational methodology, which does not allow for the establishment of causal relationships or the examination of the described processes in depth, as would experimental and qualitative methodologies. It is therefore proposed that future studies address this aspect from the above-mentioned methodological approaches. It would be advisable for future research to address all these limitations and to study similar models with experimental and/or qualitative methodologies.

## 6. Conclusions

In conclusion, we emphasize that the results are in line with the SDT postulates, and should serve to raise awareness among PE teachers about their potential responsibility in the struggle against bullying, training in styles with AS, and avoiding exercising CS to contribute to the promotion of BPN satisfaction and students’ self-determined motivations to reduce bullying perpetration and victimization.

Considering that very few previous studies have analyzed bullying from the perspective of SDT, this paper makes a relevant contribution by explaining bullying behaviors in the context of this framework.

## Figures and Tables

**Table 1:** Descriptive statistics and correlations between the perception of the supportive style in physical education, satisfaction of the basic psychological needs in physical education, students’ self-determination and bullying behaviors.

Variables	M	SD	1	2	3	4	5	6
1. Autonomy supportive style	4.76	1.27						
2. Controlling Style	3.03	1.49	−0.06					
3. Basic psychological needs	3.60	0.78	0.61 **	−0.10 *				
4. Self-determination index	2.25	4.18	0.43 **	−0.39 **	0.56 **			
5. Bullying Perpetration	1.31	0.50	−0.17 **	0.08 *	−0.24 **	−0.20 **		
6. Victimization	1.56	0.66	−0.10 *	0.04	−0.18 **	−0.13 **	0.57 **	

* *p* < 0.05. ** *p* < 0.01.
